# Fast high quality computational ghost imaging based on saliency variable sampling detection

**DOI:** 10.1038/s41598-024-57866-6

**Published:** 2024-04-02

**Authors:** Xuan Liu, Jun Hu, Mingchi Ju, Yingzhi Wang, Tailin Han, Jipeng Huang, Cheng Zhou, Yongli Zhang, Lijun Song

**Affiliations:** 1 College of Electronic and Information Engineering, Changchun University of Science and Technology, Changchun, 130022 China; 2 College of physics, Northeast Normal University, Changchun, 130024 China; 3 Academy of Agricultural Planning and Engineering, Ministry of Agriculture and Rural Affairs, Beijing, 100125 China; 4 Changchun Institute of Technology, Changchun, 130103 China

**Keywords:** Imaging and sensing, Displays, Information theory and computation

## Abstract

Fast computational ghost imaging with high quality and ultra-high-definition resolution reconstructed images has important application potential in target tracking, biological imaging and other fields. However, as far as we know, the resolution (pixels) of the reconstructed image is related to the number of measurements. And the limited resolution of reconstructed images at low measurement times hinders the application of computational ghost imaging. Therefore, in this work, a new computational ghost imaging method based on saliency variable sampling detection is proposed to achieve high-quality imaging at low measurement times. This method physically variable samples the salient features and realizes compressed detection of computational ghost imaging based on the salient periodic features of the bucket detection signal. Numerical simulation and experimental results show that the reconstructed image quality of our method is similar to the compressed sensing method at low measurement times. Even at 500 (sampling rate $0.76\%$) measurement times, the reconstructed image of the method still has the target features. Moreover, the $2160\times 4096$ (4K) pixels ultra-high-definition resolution reconstructed images can be obtained at only a sampling rate of $0.11\%$. This method has great potential value in real-time detection and tracking, biological imaging and other fields.

## Introduction

Ghost imaging (GI) is a new optical imaging technology that is different from traditional optical imaging methods. The traditional method usually uses an array detector with spatial resolution to directly obtain the target information, while GI uses a dual optical path system with one single-pixel detector. The modulated light field of the reference optical path is collected by a detector with spatial resolution, and the light intensity information of the target light path is measured by a single-pixel detector^1,2^. To make the system easier, Shapiro^3^ proposed the theory of computational ghost imaging (CGI), which uses a spatial light modulation device to replace the reference optical path and simplifies the optical structure of GI. Subsequently, Bromberg^4^ experimentally confirmed the feasibility of the CGI scheme and promoted the development of GI. Recently, GI has a wide range of applications in many fields, such as lidar imaging^5–7^, terahertz imaging^8–10^, X-ray imaging^11–14^, spectral imaging^15–18^, microscopic imaging^19–23^, etc. However, poor imaging quality and long measurement time limit the application and development of GI.

Spatial light field design and optimization is one of the effective means to improve imaging quality and reduce measurement consumption. At present, the commonly used spatial light field modulation matrices mainly include random matrices and orthogonal matrices. The random matrices need to be oversampled to obtain a reconstructed image close to the original image, and the orthogonal matrices need to be completely sampled to completely restore the target image. Both of these matrices cannot obtain high-quality reconstructed images at low measurement times. So researchers have carried out a lot of research on the optimization of the light field modulation matrix in order to obtain better target image information.

In terms of random matrix optimization, methods such as multi-scale^24–26^ , orthogonalization^27^, sparse constraints^28,29^, and singular value decomposition^30,31^ have been proposed one after another, greatly improving imaging quality and algorithm performance. However, their computational and implementation complexity is still high, and further in-depth optimization is still needed^32–34^. In terms of orthogonal matrix optimization, orthogonalization light field has attracted much attention due to its orthogonality which can perfectly restore the target image. The screening and sequence of orthogonalized light field mainly focus on the optimization of Hadamard basis^35–37^, Fourier basis^38–41^, wavelet basis^42^, etc. Thanks to the proposal of the “Russian doll” Hadamard optimization sequence^43^, a series of Hadamard optimization work has been carried out^44^, especially the combination of “cake cutting” sequence and TVAL3 compressed sensing algorithm^45^ , which greatly reduces the number of measurements while ensuring imaging quality. And, the Fourier compression ghost imaging method based on random sampling can randomly sample the low-frequency and high-frequency information of the target image to obtain high-quality reconstructed images^46,47^. However, these methods still suffer from some disadvantages, such as the limited improvement of reconstructed image quality at low measurement times, and the reliance on complex reconstruction algorithms for high-quality reconstructed images.

Currently, most GI research focuses on low-resolution imaging. As far as we know, the research of ultra-high-definition resolution GI is rarely mentioned. However, some fields such as remote sensing imaging, medical imaging, etc., need high-resolution imaging, which limits the application of GI. Hence, in order to improve the imaging quality of GI at low measurement times and realize fast ultra-high-definition resolution GI, we propose a new CGI method based on saliency variable sampling detection. According to the orthogonality of trigonometric functions, we design a sinusoidal intensity modulation patterns to obtain high-quality reconstructed images at low measurement times. Moreover, we have discovered a new phenomenon that the bucket detection signal has salient periodic features. In order to explore the reasons for the salient features, the theoretical derivation and physical explanation are conducted. And the random weight function and salient features are used to construct variable sampling windows, collecting salient information and non-salient information of the target at the same time, so that a higher quality reconstructed image can be obtained with only a few measurement times. The successful implementation of our method confirms that this method can obtain reconstructed image quality similar to the compressed sensing algorithm, improve the imaging efficiency of CGI, achieve fast ultra-high-definition resolution CGI, and promote the application of real-time CGI.

## Results

### Performance evaluation

To test the effectiveness of our method, we introduce PSNR and SSMI to evaluate the quality of reconstructed CGI images. Naturally, the larger the PSNR and SSIM values, the better the reconstructed image quality of CGI. PSNR^48^ and SSIM^49^ are defined as

1 $$\begin{aligned} PSNR = 10 \times log_{10}\left[\frac{maxVal^{2}}{MSE}\right], \end{aligned}$$

where, $MSE=\frac{1}{r\times c}\sum _{i=1}^r\sum _{j=1}^c[u(i,j)-x(i,j)]^{2}$, *maxVal* is the maximum possible pixel value of the image .

2 $$\begin{aligned} SSIM(u,x) = \frac{(2\mu _{u}\mu _{x} + C_{1})(\sigma _{ux} + C_{2})}{(\mu _{u}^{2}+\mu _{x}^{2}+C_{1})(\sigma _{u}^{2}+\sigma _{x}^{2}+C_{2})}, \end{aligned}$$

here, *u* represents the target image ($r\times c$ pixels), *x* is the reconstructed image of CGI. $\mu _{u}$, $\mu _{x}$ are the mean values of *u* and *x*, respectively. $\sigma _{u}$ and $\sigma _{x}$ are the standard deviations of *u* and *x*, respectively. And $\sigma _{ux}$ represents the cross correlation of *u* and *x*. $C_{1}$ and $C_{2}$ are constants.

### Numerical simulation

In order to confirm the effectiveness of our method, we performed numerical simulations in different scenarios of application.

**Figure 1:**
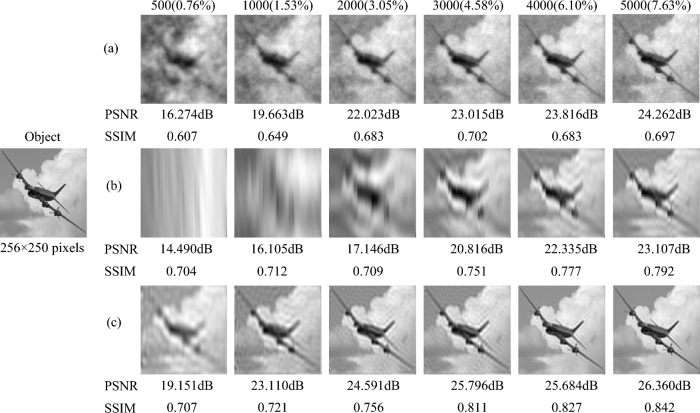
The numerical simulation results of CGI for the sparse scene in the sky at different measurement times. (**a**) is the numerical simulation result of CGI based on random pattern, reconstructed by TVAL3 compressed sensing reconstruction algorithm. (**b**) and (**c**) are the numerical simulation results of the CGI reconstructed by the second-order correlation algorithm, respectively, where (**b**) is with the original sinusoidal pattern, (**c**) is based on the optimized sinusoidal pattern.

*Sparse scene in the sky.* To simulate this scenario, we use an airplane in the sky as the target object to simulate. The numerical simulation result of CGI using different modulation patterns are shown in Fig. 1 at different measurement times. Since the ${\textbf {A}}^{T}{} {\textbf {A}}$ matrix is not a strict identity matrix, it is necessary to remove some columns of the sinusoidal pattern image to make the ${\textbf {A}}^{T}{} {\textbf {A}}$ matrix close to the identity matrix. Therefore, in order to improve the quality of the reconstructed image, we adjust the size of the sinusoidal pattern to $256 \times 250$ pixels. And the image size of the object is also $256 \times 250$ pixels. Fig. 1a is reconstructed with random pattern using TV-based CGI (TVAL3)^50^ compressed sensing algorithm. Figure 1b and c are reconstructed by the second-order correlation algorithm using the original sinusoidal pattern and the optimized sinusoidal pattern, respectively. From Fig. 1, we can find that the reconstructed image using the optimized sinusoidal pattern is clearer than the random pattern and the original sinusoidal pattern. When the number of samples is 4000 (sampling rate $6.10\%$, the sampling rate is omitted in the below.), the reconstructed image of the optimized sinusoidal pattern can clearly restore the target image, but the reconstructed images of the random pattern and the original sinusoidal pattern are blurry. Even when the number of samples is as low as 500 ($0.76\%$), the reconstructed image of the optimized sinusoidal pattern can still see the outline of the airplane. In order to compare the numerical simulation results of different methods more accurately, we calculated the PSNRs and SSIMs of the reconstructed images at different measurement times. It can be found from Fig. 1 that both the PSNR values and SSIM values of the reconstructed image gradually increase with the increase of the number of samples. And the PSNR and SSIM values of Fig. 1c are higher than Fig. 1a and b. When the number of samples is only 1000 ($1.52\%$), the PSNR value of the optimized sinusoidal pattern has exceeded 20dB, reaching 23.110dB. And when the number of samples exceeds 3000 ($4.58\%$), the SSIM value of the optimized sinusoidal pattern surpasses 0.8. Therefore, our method can obtain high-quality CGI reconstruction results for the sparse scene in the sky at low measurement times.

**Figure 2:**
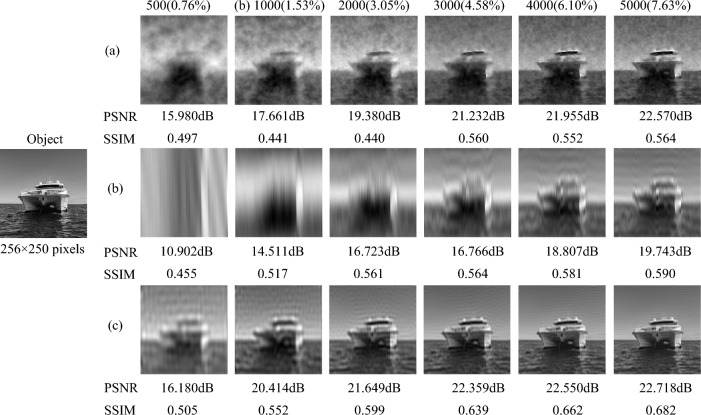
The numerical simulation results of CGI for the sparse scene at sea at different measurement times, where PSNRs and SSIMs are listed below the corresponding images. (**a**) is the simulation result using TVAL3 compressed sensing algorithm with random pattern. (**b**) and (**c**) are the simulation results with the second-order correlation algorithm based on the original sinusoidal pattern and the optimized sinusoidal pattern, respectively.

*Sparse scene at sea,* such as a ship at sea [see Fig. 2 Object image]. The size of the object image is $256 \times 250$ pixels. Fig. 2 shows the simulation result of CGI for the sparse scene at sea with random patterns, the original sinusoidal pattern and the optimized sinusoidal pattern, respectively. And the corresponding PSNRs and SSIMs are listed below each reconstruction result. The result in Fig. 2a is reconstructed by the TVAL3 compressed sensing algorithm, while Fig. 2b and c are reconstructed by the second-order correlation algorithm. It can be seen from Fig. 2 that when the number of samples is 500 ($0.76\%$), the reconstructed image of the optimized sinusoidal pattern already contains the main contour information of the ship, while the reconstructed images of the random pattern and the original sinusoidal pattern does not have the information of the ship. When the number of samples reaches 1000 ($1.52\%$), the PSNR value of the optimized sinusoidal pattern has exceeded 20dB, while the PSNR values of the random pattern and the original sinusoidal pattern are only 17.661dB and 14.511dB. The optimized sinusoidal pattern simulation results can restore the target image information well, when the number of samples is 3000 ($4.58\%$). Thus, our method has been verified to achieve high-quality CGI reconstruction of the sea target with low number of samples.

**Figure 3:**
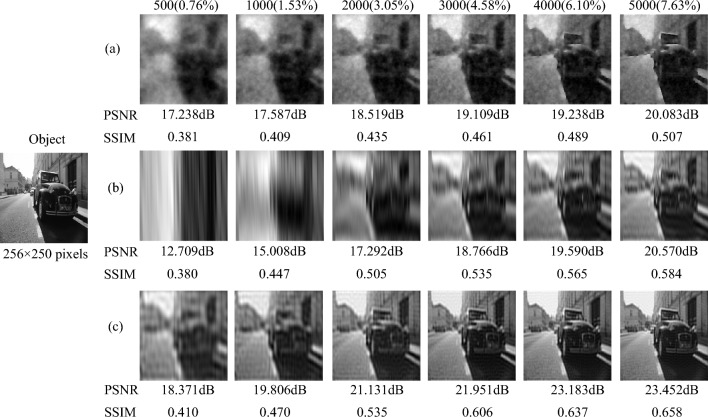
The numerical simulation results of CGI with the random pattern, the original sinusoidal pattern and the optimized sinusoidal pattern for the complex scene on the road at different measurement times, where PSNRs and SSIMs are presented together. (**a**) is reconstructed by TVAL3 compressed sensing algorithm, (**b**) and (**c**) are reconstructed by second-order association algorithm.

*Complex scene on the road.* We use a car, a street and some buildings as the target object ($256 \times 250$ pixels) to simulate the complex road scene, which is shown in Fig. 3. The simulation results and corresponding PSNRs and SSIMs in Fig. 3 are obtained with the number of samples from 500 ($0.76\%$) to 5000 ($7.63\%$). The numerical simulation result reconstructed by the TVAL3 compressed sensing algorithm based on the random pattern is displayed in Fig. 3a. The results in Fig. 3b and c are based on the original sinusoidal pattern and the optimized sinusoidal pattern respectively, reconstructed by the second-order correlation algorithm. Since the scene in Fig. 3 is more complex than those in Figs. 1 and 2, we can find that the reconstruction result of CGI based on compressed sensing random pattern is poorer. However, the complexity of the scene has relatively little influence on the reconstruction results of CGI with the original sinusoidal pattern and the optimized sinusoidal pattern. When the number of samples is 3000 ($4.58\%$), the object detail information in the simulation result of the optimized sinusoidal pattern is very clear, while the random pattern and the original sinusoidal pattern cannot obtain clear detailed information even when the number of samples is 5000 ($7.63\%$). The PSNR value of the optimized sinusoidal pattern has exceeded 20dB when the number of samples is 2000 ($3.05\%$), while the PSNR values of the random pattern and the original sinusoidal pattern only reach 20dB when the number of samples is 5000 ($7.63\%$). Even when the number of samples is 500 ($0.76\%$), the contour and position information of the car can be obtained by optimizing the sinusoidal pattern. Therefore, the simulation results show that our method can also obtain high-quality reconstructed images for the complex scene at low measurement times.

**Figure 4:**
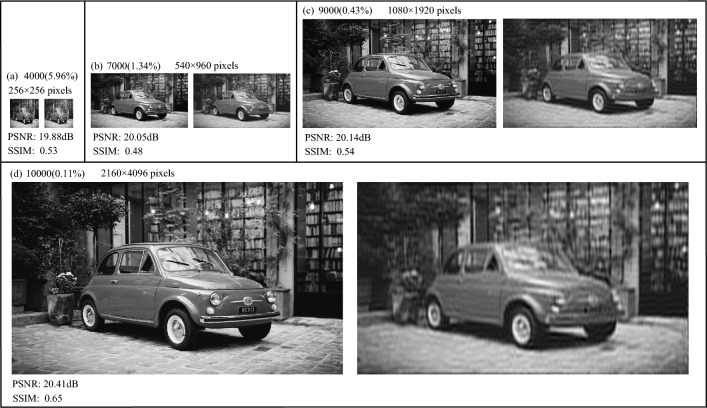
The numerical simulation results of CGI with different imaging resolutions, where PSNRs and SSIMs are presented together. In figures (**a**–**d**), the left images are the object images and the others are the reconstructed images. The resolutions of the reconstructed images in (**a**–**d**) are $256\times 256$ pixels, $540\times 960$ pixels, $1080\times 1920$ pixels, and $2160\times 4096$ pixels, respectively.

To illustrate that our method can achieve ultra-high-definition resolution CGI, the numerical simulations on the target images with different resolutions are conducted. Fig. 4 presents the results of CGI with different imaging resolutions at different numbers of samples. The left images in Fig. 4a–d are the object images with $256\times 256$ pixels, $540\times 960$ pixels, $1080\times 1920$ pixels, and $2160\times 4096$ pixels, respectively. And the right images are the corresponding reconstructed images. To improve imaging quality, we remove part of the column data for the sinusoidal intensity modulation pattern, and the arbitrary pixel-size imaging of CGI can be achieved. Fig. 4a is result of CGI with $256\times 256$ pixels imaging resolution at the 4000 ($5.96\%$) measurement times, and the PSNR and SSIM are 19.88dB and 0.53, respectively. In Fig. 4b, when the imaging resolution is increased to $540\times 960$ (540p) pixels, the sampling rate is reduced to $1.34\%$, and PNSR and SSIM are close to Fig. 4a. When the imaging resolution is further increased to $1080\times 1920$ (1080p) and $2160\times 4096$ (4K) pixels, the PSNR and SSIM values both improve. The PSNR and SSIM values of the $1080\times 1920$ (1080p) pixels reconstructed image are 20.14dB and 0.54 respectively, when the number of samples is 9000 ($0.43\%$). And when the size of the reconstructed image is $2160\times 4096$ (4K) pixels ultra-high-definition resolution, the PSNR and SSIM values increase to 20.41dB and 0.65 respectively at the 10000 ($0.11\%$) measurement times. From Fig. 4c,d, we can find that the reconstructed images are clear and contains many details. Finally, we can conclude that as the imaging resolution of CGI increases, the sampling rate gradually decreases. The simulation result confirms that our method can obtain high-quality ultra-high-definition resolution reconstructed images at low measurement times.

The numerical simulations under different noise levels were performed to illustrate the anti-noise performance of our method. We use the signal-to-noise ratio (SNR) of the bucket detection signal to measure the noise level. And add Gaussian white noise to the bucket detection signal. The signal-to-noise ratio is expressed as:

3 $$\begin{aligned} SNR=10\log _{10}\frac{B_{s}}{B_{n}} \end{aligned}$$

where, $B_{s}$ and $B_{n}$ represent the effective power of the bucket detection signal and noise, respectively.

**Figure 5:**
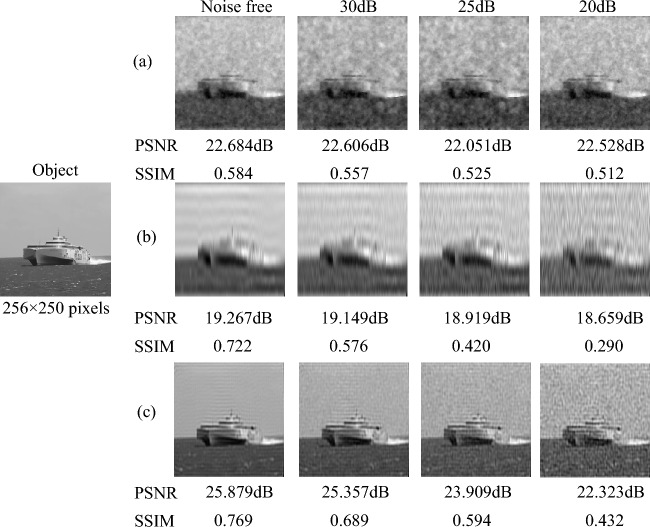
The simulation results of the proposed method under different noise levels with 4000 (6.10%) measurement times. The images in (**a**, **b**) and (**c**) are reconstructed by the random pattern, the original sinusoidal pattern and the optimized sinusoidal pattern.

It can be observed from Fig. 5 that the algorithm with the best anti noise performance is the TVAL3 compressed sensing reconstruction algorithm based on random speckle pattern. As the noise increases, the PSNR and SSIM values are very close around 22dB and 0.5 respectively. However, the quality of the results with the original sinusoidal pattern and the optimized sinusoidal pattern gradually decreases as the noise increases, and the anti-noise performance is not as good as the random speckle method based on TVAL3 compressed sensing algorithm with the random speckle pattern.

### Experimental

**Figure 6:**
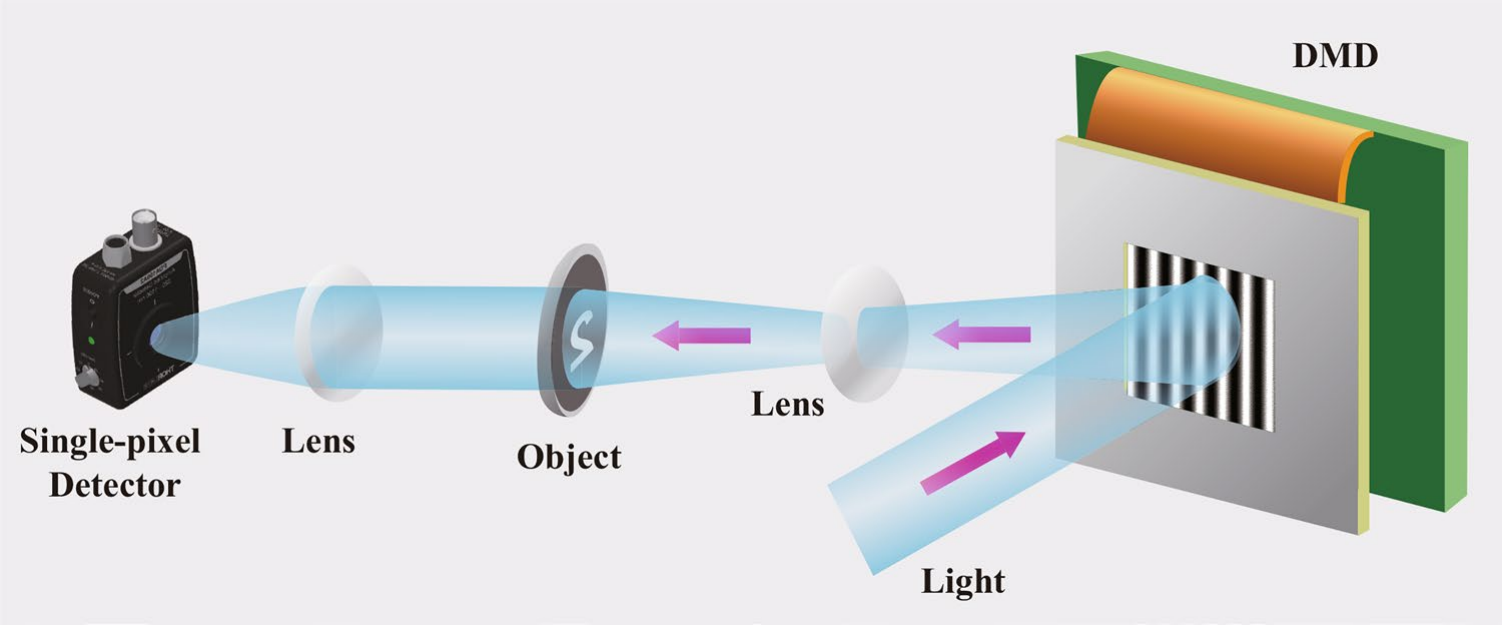
The experiment system diagram of CGI.

In order to verify the feasibility of our method, the actual CGI experiment is conducted. The experiment system is illustrated in Fig. 6, which includes a collecting lens, an imaging lens, a reflecting mirror, a digital micro-mirror device(DMD) and a single-pixel detector. The DMD is a device in a pixel multiplexing modulation scheme consisting of $1024\times 768$ micro-mirrors, and each micro-mirror can be switched in two directions of $\pm 12^\circ$, corresponding to 1 and 0. The DMD can display a preloaded sequence of sinusoidal patterns ($256 \times 250$ pixels) at speeds up to 22K patterns/s. In order to load the sinusoidal patterns into the DMD, the sinusoidal patterns are binarized by dithering^51^. Under ambient illumination (Thorlabs MCWHLP1 cold white LED ), the echo signal reflected by the mirror is collected by the single-pixel detector (Thorlabs PDA100-A2). The target object is a digital mask model of size 1 cm $\times$ 0.7 cm (see Fig. 6 Object).

**Figure 7:**
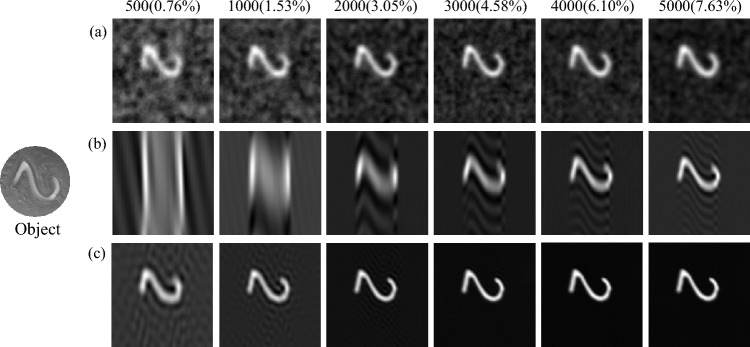
Static digital experimental reconstructed results with different measurement times. (**a**) is the experimental result reconstructed by TVAL3 compressed sensing algorithm based on the random pattern. And (**b**) and (**c**) are the experimental results reconstructed by the second-order correlation algorithm using the original sinusoidal pattern and the optimized sinusoidal pattern, respectively.

*Static digital experiment of CGI.* The reconstructed target object is a digital mask model (see Fig. 7 Object). And the static experimental reconstructed results of CGI with sample numbers from 500 ($0.76\%$) to 5000 ($7.63\%$) are shown in Fig. 7 at the DMD modulation rate of 20 KHz. In Fig. 7 all reconstructed images are $256 \times 250$ pixels. Fig. 7a–c are the experimental results with the random pattern, the original sinusoidal pattern and the optimized sinusoidal pattern, respectively. Fig. 7a is reconstructed by TVAL3 compressed sensing algorithm, and Fig. 7b,c are reconstructed by second-order correlation algorithm. From Fig. 7a–c, it can be observed that the results obtained with the optimized sinusoidal pattern are better than those obtained with the random pattern and the original sinusoidal pattern. Especially, when the measurement times are 500 ($0.76\%$), the reconstructed targets of the random pattern and the original sinusoidal pattern cannot be clearly distinguished, while the reconstructed target of the optimized sinusoidal pattern is still clear. When the measurement times increase to 3000 ($4.58\%$), the optimized sinusoidal pattern can perfectly restore the target image. The experimental result confirms that our method is feasible to obtain high-quality reconstructed images at low measurement times.

**Figure 8:**
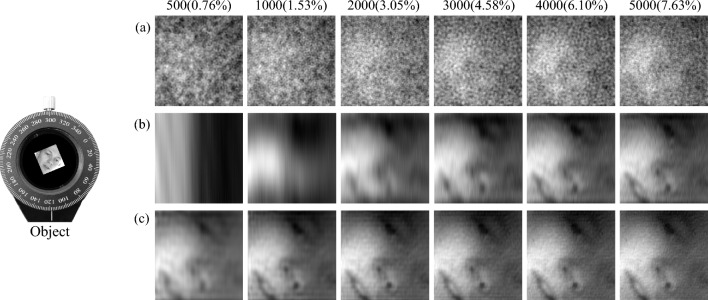
Static complex experimental reconstructed results with different measurement times. (**a**) is the experimental result reconstructed by TVAL3 compressed sensing algorithm based on the random pattern. And (**b**) and (**c**) are the experimental results reconstructed by the second-order correlation algorithm using the original sinusoidal pattern and the optimized sinusoidal pattern, respectively.

*Static complex experiment of CGI.* The reconstructed target object is a human face (see Fig. 8 Object) of BioID dataset. And the experimental result is as shown in Fig. 8, where the image pixel size is $256 \times 250$ pixels and the number of measurements ranges from 500 to 5000. It can be found from Fig. 8 that the results obtained using the optimized sinusoidal pattern are the best. Even with 500 measurement times, the target’s face image can be obtained, while neither random pattern nor the original sinusoidal pattern can see the face features. As the measurement time increases to 2000, the facial information can be seen in the original sinusoidal pattern, while random pattern still have no facial features. Experimental results confirm that our method can achieve high-quality imaging of complex targets at low measurement times.

**Figure 9:**
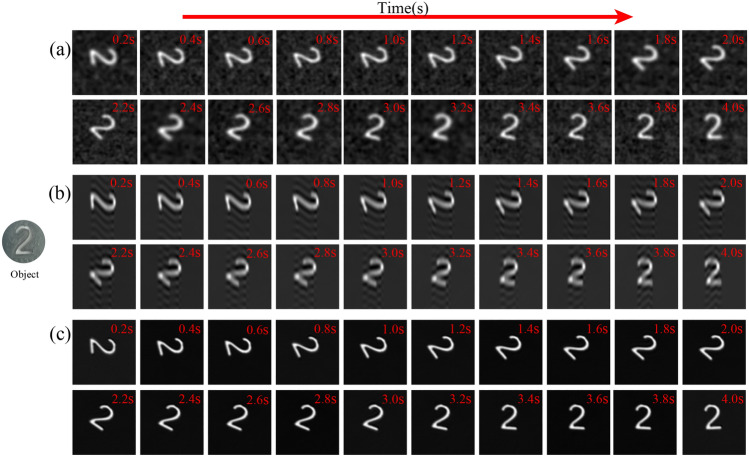
Dynamic experimental reconstructed results with 4000 ($6.10\%$) measurement times at a DMD modulation rate of 20 KHz for 4 seconds. (**a**, ** b**), and (**c**) are the reconstruction results of the CGI with the random pattern, the original sinusoidal pattern, and the optimized sinusoidal pattern, respectively.

*Dynamic experiment of CGI.* To illustrate the performance of the method on a moving target, the CGI experiment with a duration of 4s was performed on the rotating target object (see Fig. 9) with 4000 ($6.10\%$) sampling numbers at the DMD modulation rate of 20 KHz. The size of the reconstructed image is $256 \times 250$ pixels. And the experimental result is shown in Fig. 9. As shown in Fig. 9, the rotation direction of the object is counterclockwise, and the imaging frame rate of CGI is calculated at 5 frames per second. The result in Fig. 9a is reconstructed by the TVAL3 compressed sensing algorithm with the random pattern. Fig. 9b and c are the results of the second-order correlation reconstruction using the original sinusoidal pattern and the optimized sinusoidal pattern, respectively. It can be found that the quality of the images in Fig. 9c is significantly better than Fig. 9a and b. Specifically, the background noise of the result in Fig. 9a is large, and the resolution of the results in Fig. 9b is lower, while the results in Fig. 9c have great advantages in terms of quality and resolution. The experimental result proves that our method can obtain high-quality reconstructed images under undersampling, and can achieve real-time high-quality computational CGI at 5 frames per second. Meanwhile, it can be predicted that our method can achieve fast real-time target tracking at a speed of 40 frames per second under 500 ($0.76\%$) measurement times, which has certain potential application value in this field.

## Discussion and conclusions

In this paper, we proposed a new method for fast high quality CGI system based on saliency variable sampling detection. This method utilizes the characteristics of the salience characteristics of the barrel detection signal to optimize the sequence of sinusoidal intensity modulation speckle sequences. A variable sampling CGI method based on salient feature detection and supplemented by non-saliency detection was implemented.

A detailed characterisation of the developed fast high quality CGI method was presented. First, we obtained high-quality reconstructed images with low measurement times by using the sinusoidal intensity modulation light field. Then, we found that the bucket detection signal has a saliency periodic features by studying the spectrum of the sinusoidal intensity modulation sequence. So the target image can be variably sampled using the optimized sinusoidal intensity modulation sequence, which is reordered through a random weight function to simultaneously obtain saliency information and non-salient information of the target image with low measurement times. The numerical simulation and experimental results show the effectiveness and advancement of this method. It can not only get high-quality reconstructed images at low measurement times, but also obtain reconstructed images that are better than compressed sensing at the same measurement times. In addition, this work provides the possibility for fast ultra-high-definition resolution CGI, and has potential application value in real-time detection and tracking, biological imaging and other imaging fields.

## Methods

### CGI reconstruction method

In a CGI system, the transmitted beam is modulated onto an object with a transmission coefficient of *T*(*i*, *j*) (the size is $r\times c$) through a spatial light modulation device, where, $i=1,2,3,\ldots ,r$, $j=1,2,3,\ldots ,c$. The total echo signal is collected by a single-pixel detector, and the collected value of *m*th sampling is recorded as $B^{(m)}=\sum _{i=1}^r\sum _{j=1}^c I^{(m)}(i,j)T(i,j)$, where $I^{(m)}(i,j)$ represents the light field of the *m*th modulated by the spatial light modulation device. The target object can be obtained by computing the correlation between $I^{(m)}(i,j)$ and $B^{(m)}$:

4 $$\begin{aligned} O(i,j) = \langle B^{(m)}I^{(m)}{(i,j)}\rangle , \end{aligned}$$

where, *m* is the number of measurements, $m=1,2,3,...,M$, $\langle \cdot \rangle =\frac{1}{M}\sum _{m=1}^M(\cdot )$.

After *M* measurements, we can convert each light field $I^{(m)}(i,j)$ into an $M\times N$ matrix *A*:

5 $$\begin{aligned} {\textbf {A}} = \begin{bmatrix} I^{(1)}(1,1) &{} I^{(1)}(1,2) &{} \cdots &{} I^{(1)}(r,c) \\ I^{(2)}(1,1) &{} I^{(2)}(1,2) &{} \cdots &{} I^{(2)}(r,c) \\ \vdots &{} \vdots &{} \ddots &{} \vdots \\ I^{(M)}(1,1) &{} I^{(M)}(1,2) &{} \cdots &{} I^{(M)}(r,c) \\ \end{bmatrix}, \end{aligned}$$

Thus, equation (4) can be expressed in matrix form as:

6 $$\begin{aligned} {\textbf {O}} = \frac{1}{M}{} {\textbf {A}}^{T}{} {\textbf {A}}{} {\textbf {T}}, \end{aligned}$$

where, ${\textbf {T}}$ is a one-dimensional column vector of dimension $N \times 1$ which is reshaped by the target object *T*(*i*, *j*). ${\textbf {O}}$ is the reconstruction result of the CGI, which is also a one-dimensional column vector with dimension $N \times 1$.

**Figure 10:**
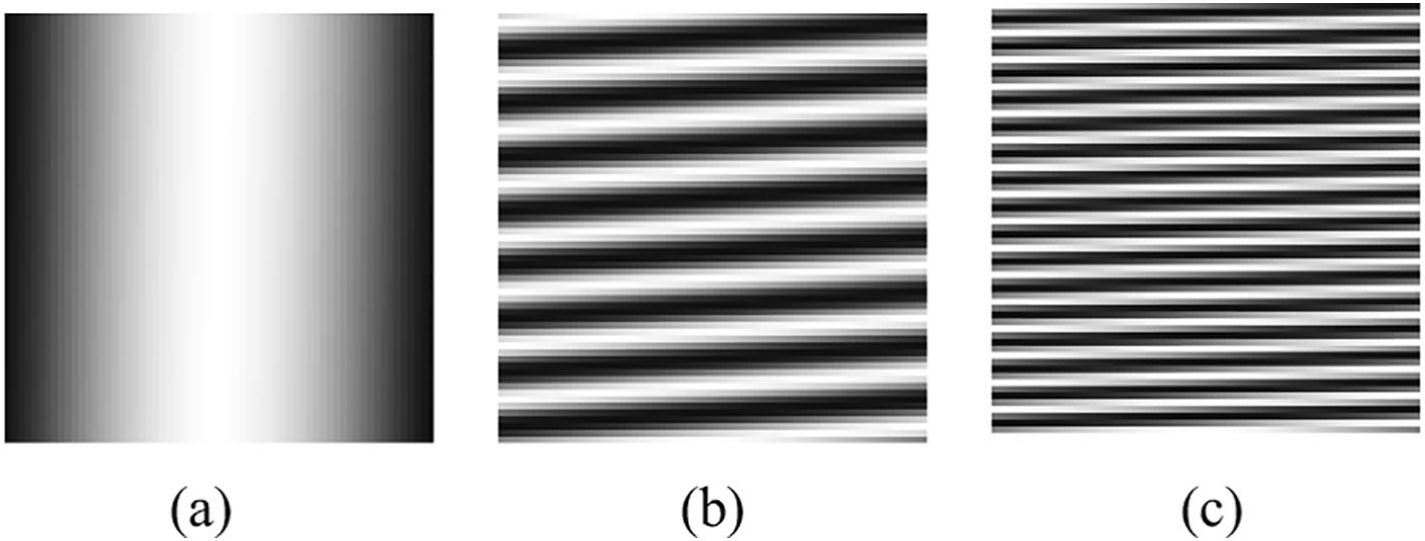
Sinusoidal intensity modulation patterns. (**a**) is the sinusoidal intensity modulation pattern at $M = 0$. (**b**) is the sinusoidal intensity modulation pattern at $M = 1024$. (**c**) is the sinusoidal intensity modulation pattern at $M = 2048$.

Through the analysis of Equation (6), it can be found that when ${\textbf {A}}^{T}{} {\textbf {A}}$ is closer to the identity matrix, the reconstructed result is closer to the target object. Here, we proposed a new method for CGI using sine function for light intensity modulation. Since any two functions of different frequency in the trigonometric function system are orthogonal, the high-quality reconstruction result can be obtained by using the sine function as the measurement matrix of the CGI. The sine function matrix is defined as:

7 $$\begin{aligned} {\textbf {A}}= & {} a\cdot sin[\frac{m\pi x}{N} + \frac{(m\times n)\pi y}{N}], \nonumber \\{} & {} (m=1,2,\cdots ,M; n=1,2,\cdots ,N.) \end{aligned}$$

where, *a* is the amplitude constant. The Sinusoidal intensity modulation patterns generated by the sinusoidal function matrix at different measurement times *M* is shown in Fig. 10. And Fig. 10a–c are the sinusoidal intensity modulation patterns when *M*=0, 1024 and 2048 respectively.

**Figure 11:**
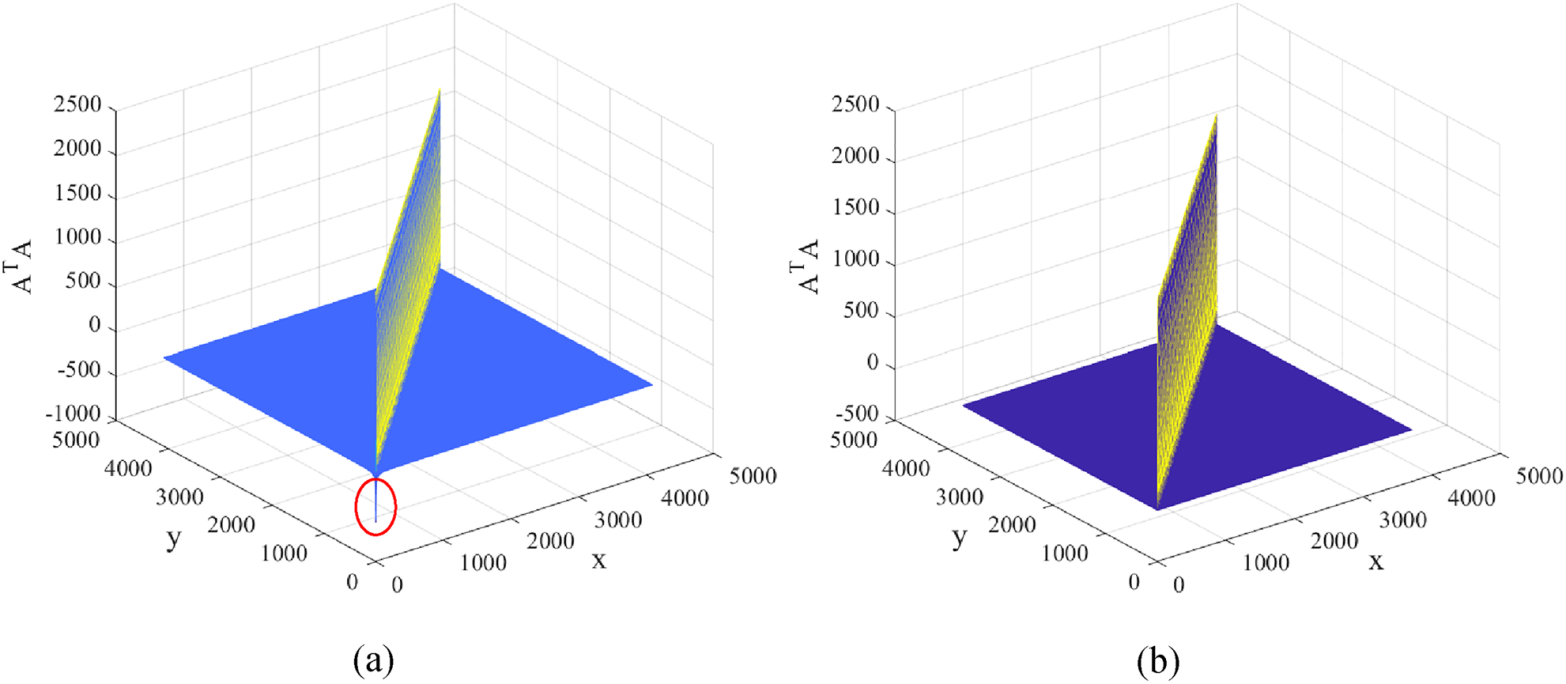
The results for ${A}^{T}{A}$. (**a**) is the result for ${A}^{T}{A}$ of $64\times 64$ pixels size sinusoidal pattern. (**b**) is the result for ${A}^{T}{A}$ of $64\times 58$ pixels size sinusoidal pattern.

To illustrate our proposed light field modulation matrix is orthogonal, the ${A}^{T}{A}$ value of the sinusoidal intensity modulation matrix is calculated, which is shown in Fig. 11. The ${A}^{T}{A}$ calculation result of the sine function matrix A is presented in Fig. 11a. The dimension of A is $4096\times 4096$, that is, the size of the sinusoidal pattern is $64\times 64$ pixels. From Fig. 11a, it can be observed that there are some outliers in the values of ${A}^{T}{A}$ non-diagonal elements in the sinusoidal pattern, which will reduce the quality of the reconstructed image. In order to improve the quality of the reconstructed image, we removed part of the column data of the sinusoidal pattern and obtained the pattern of $64\times 58$ pixels. Fig. 11b displays the result for ${A}^{T}{A}$ of $64\times 58$ pixels sinusoidal pattern. As observed, the values of ${A}^{T}{A}$ non-diagonal elements in $64\times 64$ pixels sinusoidal pattern are larger than those in $64\times 58$ pixels sinusoidal pattern. Therefore, it is necessary to remove part of the column data of the sinusoidal pattern to improve the quality of the reconstructed image.

### Saliency variable sampling detection method

Although high-quality CGI reconstruction results can be obtained by using the sinusoidal measurement matrix, the quality of the reconstructed image cannot achieve the expected results when the number of measurements is very low. In order to obtain high-quality CGI reconstruction results, we explored the sinusoidal intensity pattern sequence and proposed an optimization method for the sinusoidal intensity modulation pattern sequence based on saliency variable sampling detection. Our method allows the optimized sinusoidal intensity modulated pattern to obtain most of the information of the target with fewer samples, thereby achieving high-quality image reconstruction.

**Figure 12:**
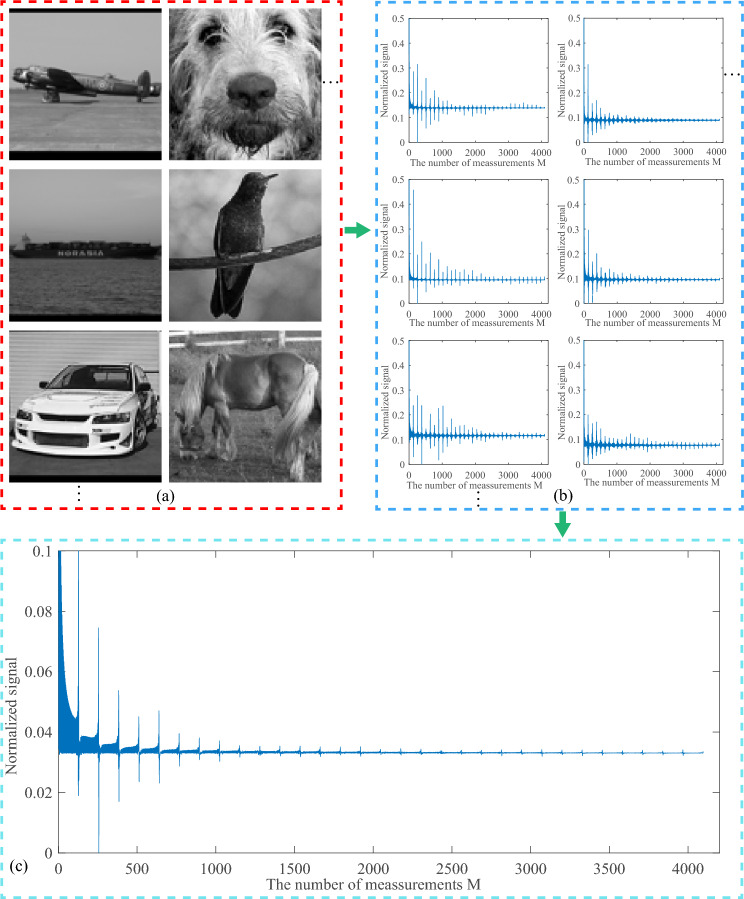
The statistical analysis results of bucket detection signals for 500 natural images. (**a**) is the 500 target images. (**b**) is the bucket detection signals normalized to (0, 1) corresponding to 500 target images. And (**c**) is the average of the 500 bucket detection signals normalized to (0, 1) in (**b**), which is normalized. In order to show the details of the bucket detection signals in (**b**) and (**c**), their vertical axes are partially enlarged, respectively.

*Statistical analysis of the bucket detection signal based on sinusoidal intensity modulation.* InCGIred by the work of Sun et al.^43^, the bucket detection signal is related to the amount of information of the target object. So we performed a statistical analysis of the bucket detection signal. Specifically, we use STL-10 database^52^, which provides different categories of natural images. And we used 500 images of different categories for statistical analysis. They are all grayscaled and resized to $64 \times 64$ pixels. Fig. 12 is the result of statistical analysis. Fig. 12a shows the 500 target images of different categories. And the normalized bucket detection signals of these target images are shown in Fig. 12b. Then, we averaged all the bucket detection signals and normalized the averaged signal, which is shown in Fig. 12c. From the bucket detection signals in Fig. 12b, we can find that the bucket detection signals of different categories of images all have similar salient periodic features. Fig. 12c shows that the salient features of the bucket detection signal obtained by the CGI method based on sinusoidal intensity modulation has a statistical regularity. It can also be found from Fig. 12c that the number of cycles of the bucket detection signal (the target image of $64 \times 64$ pixels) is 32, and each cycle has 128 sampling points.

**Figure 13:**
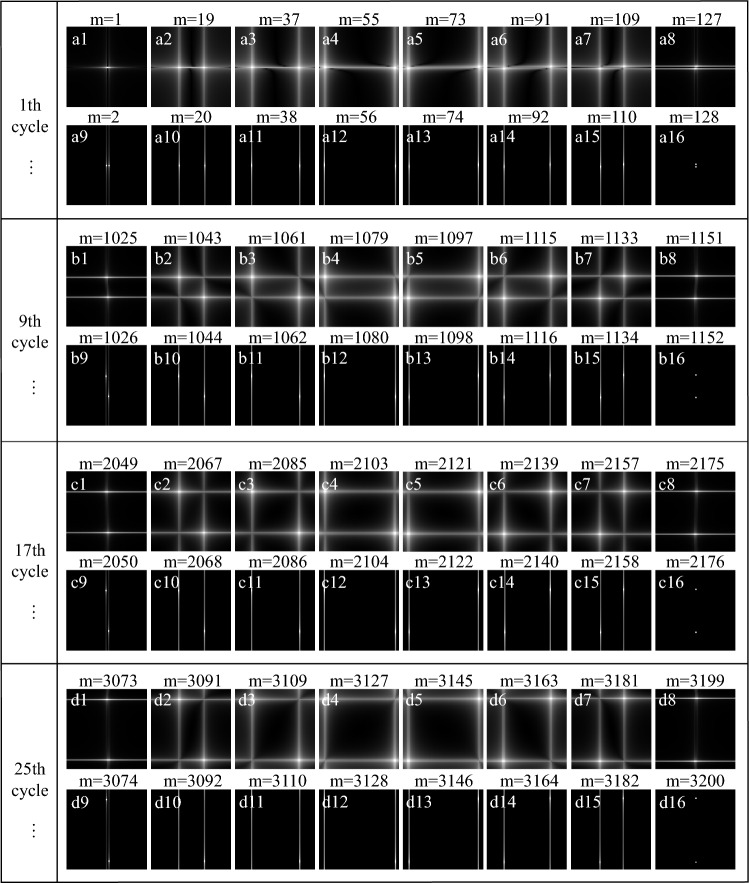
The amplitude spectrum of the sinusoidal intensity modulation pattern, where *m* is the number of samples. (**a**–**d**) represent the amplitude spectrum of different periods, respectively. (**a1**–**a8**) represent the amplitude spectrum when the measurement times *m* is odd, and (**a9**–**a16**) represent the amplitude spectrum when the measurement times *m* is even. (**b1**–**b16**), (**c1**–**c16**) and (**d1**–**d16**) are similar to (**a1**–**a16**).

*Frequency spectrum analysis of sinusoidal intensity modulation pattern.* In order to optimize the sinusoidal intensity modulation pattern sequence based on the salient periodic features of the bucket detection signal, the 2D Fourier magnitude spectrum of the patterns were analyzed, which is shown in Fig. 13. And it consists of a total of 32 cycles, with each cycle containing 128 patterns. Fig. 13a–d are the amplitude spectrum of different periods, where Fig. 13a represents the amplitude spectrum of the first cycle, Fig. 13b is the amplitude spectrum of the 9th and cycle, Fig. 13c is the amplitude spectrum of the 17th cycle, and Fig. 13d is the amplitude spectrum of the 25th cycle. From Fig. 13a1–a16, it can be found that the amplitude spectrum of the sinusoidal intensity modulation pattern is related to the parity of the sampling number *m*. The amplitude spectrum is different when *m* takes different parity values. Fig. 13a1–a8 are the amplitude spectrum images when the measurement times *m* is odd, and Fig. 13a9–a16 represent the amplitude spectrum images when *m* is even. We can see that the amplitude spectrum images in Fig. 13a1–a8 and the amplitude spectrum images in Fig. 13a9–a16 are completely different. Although they are different, the variation law of their amplitude spectrum frequency is similar, that is, the frequency first increases and then decreases. Thus, we mainly explain the frequency variation law of odd *m*. Fig. 13a1–a8 show that as the value of *m* increases, the transverse frequency of the sinusoidal pattern are same, and the longitudinal frequency first increases and then decreases. The frequency changes also have similar law in Fig. 13b1–b8, c1–c8 and d1–d8. And from Fig. 13a1–d1, we can find that their vertical frequencies are basically the same, and their horizontal frequencies gradually increase. So we can conclude that the spectrum of the sinusoidal intensity modulation pattern is periodic. In a cycle, the frequency first increases and then decreases with the number of samples *m*. During different periods, the pattern frequency increases gradually with the increase of the number of periods.

In order to obtain high-quality reconstructed images at low measurement times, we need to study the relationship between the sinusoidal intensity modulation pattern and the bucket detection signal. According to Fourier theorem, we can use the sine function to represent the target image and the sinusoidal intensity modulation pattern respectively. The target image *T*(*i*, *j*) can be expressed as $T(i,j) = \sum _{k}a_{k}S_{k}(i,j)$, where $a_{k}$ represents the *k*th Fourier coefficient, $S_{k}$ is a single frequency sine function basis. The *m*th sinusoidal intensity modulation pattern is $I^{(m)}(i,j) = \sum _{n}b^{(m)}_{n}S_{n}(i,j)$, where $b^{(m)}_{n}$ is the *n*th Fourier coefficient, $S_{n}$ is a single frequency sine function basis. Therefore, the bucket detection signal $B^{(m)}$ can be rewritten as

8 $$\begin{aligned} B^{(m)}= & {} \sum _{i=1}^r\sum _{j=1}^c T(i,j)I^{(m)}(i,j) \nonumber \\= & {} \sum _{i=1}^r\sum _{j=1}^c (\sum _{k}a_{k}S_{k}(i,j))(\sum _{n}b^{(m)}_{n}S_{n}(i,j)) \\= & {} \sum _{k}\sum _{n} a_{k}b^{(m)}_{n}, \nonumber \end{aligned}$$

where, according to the orthogonality of the trigonometric basis, we can get:

9 $$\begin{aligned} \sum _{i=1}^r\sum _{j=1}^c S_{k}(i,j)S_{n}(i,j) = {\left\{ \begin{array}{ll} 1, &{} (k = n)\\ 0, &{}(k \ne n) \end{array}\right. } \end{aligned}$$

Equation (8) shows that the frequency of the target image collected by the single-pixel detector is related to the frequency of the sinusoidal intensity modulation pattern. When the pattern spectrum is low frequency, the data collected by the detector is the low frequency information of the target image.

**Figure 14:**
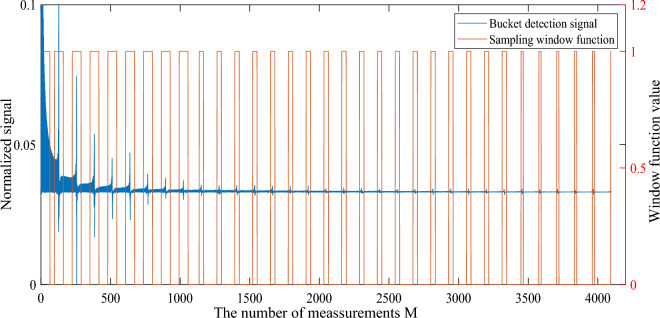
The saliency variable sampling detection method of CGI based on sinusoidal intensity modulation pattern sequence. The blue line is the bucket detection signal normalized to (0, 1), and the red line is the sampling windows function. In order to show the details of the bucket detection signal, the vertical axis is partially enlarged.

It is well known that most of the information of natural images is concentrated in the low spatial frequency band, and the sampling needs to be dominated by low frequency components and supplemented by high frequency components^17,46^. According to the spectral image of the sinusoidal intensity modulation pattern, it can be found that the salient informations (low-frequency components) are mainly located at both ends of each cycle, and the middle part is the non-salient informations (high-frequency components). Thus, we proposed an optimization method for sinusoidal intensity modulation pattern sequences based on saliency variable sampling detection, which realized high-quality CGI with low measurement times. The method of CGI is shown in Fig. 14. The normalized bucket detection signal is the blue line, and the red line is the variable sampling window function in Fig. 14. The sampling window function is a function with values only 0 and 1. When the value of the variable window function is 1, we will select the sinusoidal intensity modulation pattern corresponding to this index position. Through the sampling window function, we preferentially collect the most important part of the target image, and simultaneously obtain a large amount of low-frequency information and a small amount of high-frequency information.

Although high-quality reconstructed images can be obtained using the variable sampling method of CGI based on sinusoidal intensity modulation pattern, we cannot define the position and width of each window of the sampling window function in practical applications. To solve this problem, we propose a random weight function:

10 $$\begin{aligned} f(m) = {\left\{ \begin{array}{ll} 1, &{} m \in [1, \alpha ]\\ g_{1}(m)g_{2}(m), &{}otherwise \end{array}\right. } \end{aligned}$$

where, *m* is the number of measurements, $\alpha < M$ represents the distance from the sampling starting point with a weight of 1, and *M* is the total number of measurements. $g_{1}(m)=b^{\frac{(m-1)}{M}}$ is an exponential function. *b* is a constant. $g_{2}(m)$ is a uniformly distributed random function between 0 and 1, which can ensure that the weight value of f(m) is between 0 and 1. So $g_{1}(m)g_{2}(m)$ can ensure that the weight of some speckle patterns with large m value (mth measurement) is greater than the speckle pattern with small m value, thereby placing the speckle pattern with large m value at the front of the sequence, realizing the collection of a large number of target images. Low-frequency information and a small amount of high-frequency information collected.

**Figure 15:**
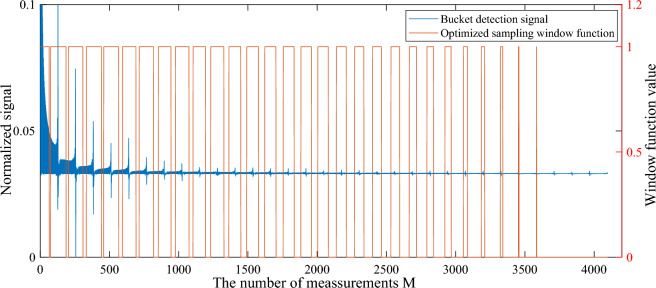
The saliency variable sampling detection method of CGI based on optimized sinusoidal intensity modulation pattern sequence. The blue line is the bucket detection signal normalized to (0, 1), and the red line is the optimized sampling windows function. In order to show the details of the bucket detection signal, the vertical axis is partially enlarged.

By equation (10), we can obtain the weight coefficients of each sinusoidal intensity modulation pattern image, and sort them in descending order. For simplicity, we take the $64 \times 64$ pixels target image as an example. Due to the frequency distribution of each cycle in the sinusoidal intensity modulation pattern sequence follows a pattern with small ends and large middle, the low frequency parts at both ends should be collected first. For computational convenience, we divide each cycle of the sinusoidal intensity modulation pattern sequence into 2 half cycles, and we can obtain the sinusoidal intensity modulation pattern sequence with a period number of 64. Then we need to determine the width of each sampling window. We first find the speckle pattern with the largest weight value, determine its m value (representing the mth measurement), and calculate the position of m in the 64 sampling windows (for example, m=1, located in the first sampling window, m=35, located in the second sampling window) , put all speckle patterns into the corresponding sampling window according to this rule. Then count the number of speckle patterns in all sampling windows to determine the width of all sampling windows. Finally, the sinusoidal speckle sequence is sorted according to the sampling window to obtain the optimized sinusoidal sequence. The variable sampling method of CGI based on optimized sinusoidal intensity modulation pattern sequence is shown in Fig. 15, where $M=2048$, $\alpha =64$, $b=0.1$.
